# Study on Tritium and Iodine Species Transport through Porous Granite: A Non-Sorption Effect by Anion Exclusion

**DOI:** 10.3390/toxics10090540

**Published:** 2022-09-16

**Authors:** Yunfeng Shi, Song Yang, Wenjie Chen, Weijia Xiong, Aiming Zhang, Zhixiang Yu, Bing Lian, Chuan-Pin Lee

**Affiliations:** 1 Department of Nuclear Environmental Science, China Institute for Radiation Protection (CIRP), Taiyuan 030006, China; 2 CNNC Environmental Protection Corporation (ECPC), Beijing 100045, China; 3 School of Nuclear Science and Engineering, East China University of Technology, Nanchang 330013, China

**Keywords:** non-sorption, anion exclusion, iodine, two-region non-equilibrium, granite

## Abstract

The safety of deep geological repositories is important in the disposal of high-level radioactive waste (HLW). In this study, advection–dispersion experiments were designed to build a transport model through a calibration/validation process, and the transport behavior of tritiated water (HTO) and various iodine species (iodide: I^−^ and iodate: IO_3_^−^) was studied on a dynamic compacted granite column. Breakthrough curves (BTCs) were plotted under various flow rates (1–5 mL/min). BTCs showed that the non-sorption effect by anion exclusion was observed only in I^−^ transport because the retardation factor (R) of I^−^ was lower than that of HTO (R = 1). Moreover, equilibrium and nonequilibrium transport models were used and compared to identify the mobile/immobile zones in the compacted granite column. The anion exclusion effect was influenced by the immobile zones in the column. The non-sorption effect by anion exclusion (R < 1) was only observed for I^−^ at 5.0 ± 0.2 mL/min flow rate, and a relatively higher Coulomb’s repulsive force may be caused by the smaller hydration radius of I^−^(3.31 Å) than that of IO_3_^−^(3.74 Å).

## 1. Introduction

It is necessary to carry out safety evaluations for deep geological disposal site of high-level radioactive waste (HLW) to evaluate the migration behavior of nuclides in surrounding rock [1]. In general, a common cause of radionuclide migration is groundwater intrusion into HLW repositories due to the failure of engineering barriers. Granite is a potential crystalline host rock in HLW geological disposal [2,3,4,5].

Considerable research efforts worldwide have increased our knowledge and understanding of how underground disposal systems function over long periods of time. Recently, several studies have investigated the advection–dispersion behavior of cationic radionuclides, such as ^238^Pu, ^237^Np, ^137^Cs, and ^90^Sr [6,7,8,9,10], to evaluate the safety of the migration of various radionuclides in deep geological environments. At present, cationic radionuclides in liquid show high adsorption ability on mineral surfaces because of the permanently negative charges by isomorphous substitution in the crystal lattice of a mineral. Migration of cationic radionuclides in host rocks would be retarded, but anionic radionuclides (i.e., ^36^Cl, ^99^Tc, ^129^I) show exclusive or repulsive behavior by Coulomb’s force [11,12,13]. Few studies on safety assessments (SA) for HLW disposal focused on the anion exclusion effect of anionic nuclide transport in granite and other host rocks [14,15,16].

In addition to transuranic radionuclides (TRUs) in HLW, the long-lived radionuclides that exhibit a relatively high mobility under deep geochemical conditions must be considered when building an HLW repository. As a primary fission product, ^129^I is characterized by a long half-life (t_1/2_ = 1.57 × 10^7^ years), high fission yield, easy volatilization, easy migration, high radioactive toxicity, high bioavailability, and weakly adsorbed radionuclide anions [17,18,19,20]. Actually, iodine species that have various valence states (iodide (I^−^), iodate (IO_3_^−^), I_2_, and organic iodine) depend on pH and redox conditions in aqueous solution. Thus, ^129^I is the main source of the potential risk posed not only by HLW repositories but also by nuclear accidents, such as the 2011 Fukushima Daiichi nuclear disaster. This threat was recently made obvious when ^129^I was observed in the Pacific Ocean around Japan, where fast dispersion by seawater movement and deposition caused it to migrate into the marine sediments [21].

The in-house dynamic column technique and numerical analyses have been simultaneously applied to simulate the transport of different contaminated solutes under natural groundwater conditions to provide a safety assessment for environmental impact on industrial or radioactive waste storage and disposal. According to different experimental conditions, the tests are designed to the filling mode of geotechnical medium (undisturbed or broken), solute injection mode (pulse or continuous injection), and water content maintenance mode (unsaturated or aquifer zone) [22,23,24]. To understand the anion exclusion effect, a previous study injected tritiated water (HTO) and anions into the same column and expressed the “accelerated” migration of anions by comparing the experimental breakthrough curves (BTCs) with the effluent concentration [25]. By comparing the BTCs of HDO and Cl^−^ in the dual-porosity structure of peat, McCarter obtained the anion exclusion of Cl^−^ and found that anion exclusion weakens with increasing Cl^−^ diffusion in pores [16]. Rao and Jain [25] determined the “accelerated” transport of [CO(CN)_6_]^−^ under the anion exclusion effect by analyzing the migration concentration distribution curves of HTO and [CO(CN)_6_]_3_^−^ in clay and established a numerical model. In addition, some studies proposed “accelerated” migration by comparing the peak position of BTCs with pore volume (PV) (if PV < 1, then accelerated transport occurs). Shukla studied the transport behavior of Cl^−^ in soil columns of different particle sizes and indicated the anion exclusion by comparing the peak position. However, the homogeneity of the filling soils must be guaranteed to prevent the preferential flow (PV < 1) in the column.

In 1972, a two-region numerical model including the immobile/mobile zone in saturated porous soil based on diffusion double layer (DDL) theory was first developed by Krupp for the solute’s pore velocity distribution formed by anion exclusion [26,27]. In addition, a physical nonequilibrium ion exclusion model was proposed and modified from DDL, and the porewater in porous soil was separated and expressed as flowing and nonflowing areas. Moreover, field experimental results by Gvirtzman [28] explained a dispersion model without ion exchange and an exchange model with ion exchange and obtained a good fitting.

In a previous work [29,30], individual and coexisting I^−^ and IO_3_^−^ were successfully analyzed by applying ion chromatography (IC) coupled with a convenient and rapid detection tool for inductively coupled plasma optical emission spectrometry (ICP-OES), and it has become a quick and effective method in analyzing anion speciation in environmental samples. In the present study, the advection-dispersion behavior of I^−^ and IO_3_^−^ in granite was studied and used to simulate their transport behavior in a micropore system. The BTCs of HTO, I^−^, and IO_3_^−^ at different flow rates were plotted and compared, and the key transport parameters were estimated from STudio of ANalytical MODels (STANMOD) and HYDRUS-1D under various conditions. The *R*, water content in the immobile regions (*θ_im_*), first-order mass transfer coefficient (α), and other parameters were obtained to quantify the anion exclusion effect, and the influence of flow rate and iodine speciation on anion exclusion was explored. The results will support safety assessments of the impact of “accelerated migration” of anionic radionuclides for HLW repositories.

## 2. Theory of Advection and Dispersion

### 2.1. Equilibrium Transport

The majority of studies focused on radionuclide transport in saturated porous media, such as compacted crushed granite, and the one-dimensional governing solute transport equation is:

(1) $$\frac{\partial C}{\partial t}=D\frac{\partial^{2}C}{\partial x^{2}}-v\frac{\partial C}{\partial x},$$

where *C* is radionuclide concentration in solution [Bq/mL^3^], v is the average pore-water velocity [cm/min], t is time [min], and *x* is the distance [cm]. *D* is the dispersion coefficient of the granite media [cm ^2^/min], and it can be further defined and written as

(2) $$D=D_{L}v+D^{*},$$

where $D_{L}\left[\mathrm{cm}\right]$ is the dispersivity and $D^{*}$ the diffusion coefficient in free water [cm^2^/min]. For the case where the adsorption equilibrium can be reached instantaneously or it takes a long time to reach the adsorption equilibrium, the distribution coefficient $K_{d}$ is expressed as

(3) $$S=K_{d}C,$$

where $K_{d}$ is the distribution coefficient [mL^3^/g]. In this study, we only take account of instant adsorption of radionuclides on the pores of crushed granite, and Equation (2) can be re-written as

(4) $$\frac{\partial C}{\partial t}+\frac{\rho_{b}}{\theta }\frac{\partial S}{\partial t}=D\frac{\partial^{2}C}{\partial x^{2}}-v\frac{\partial C}{\partial x},$$

where θ is the porosity or volumetric water content [-], and *ρ_b_* is the bulk density of porous medium [mL^3^/g]. It can be combined by a retardation factor given by $\;R=1+\frac{\rho_{b}K_{d}}{\theta }$, and Equation (3) can be rewritten as

(5) $$R\frac{\partial C}{\partial t}=D\frac{\partial^{2}C}{\partial x^{2}}-v\frac{\partial C}{\partial x}$$

In addition, the boundary conditions and initial conditions are

(6) $$C\left(x,0\right)=0,\;0<x<L$$

(7) $$C\left(0,t\right)=C_{0}\;$$

(8) $$\frac{\partial C\left(\infty ,t\right)}{\partial t}=0\;$$

where $C_{0}$ is inlet radionuclide concentration [Bq/mL^3^], and *L* is the length of the column [cm].

### 2.2. Two-Region Nonequilibrium Transport

A two-region transport model was proposed and assumed that the liquid phase can be partitioned into mobile (flowing) and immobile (stagnant) regions (which are produced by anion exclusion). The solute exchange between the two liquid regions is simulated as a first-order kinetic process. The two-region solute transport model is given by

(9) $$(\theta_{m}+f\rho_{b}K_{d})\frac{\partial c_{m}}{\partial t}=\theta_{m}D_{m}\frac{\partial^{2}c_{m}}{\partial x^{2}}-J_{w}\frac{\partial c_{m}}{\partial x}-a\left(c_{m}-c_{im}\right)-\left(\theta_{m}\mu_{l,m}+f\rho_{b}K_{d}\mu_{s,m}\right)c_{m}+\theta_{m}r_{l,m}\left(x\right)+f\rho_{b}r_{s,m}\left(x\right)$$

(10) $$(\theta_{im}+\left(1-f\right)\rho_{b}K_{d})\frac{\partial c_{im}}{\partial t}=a\left(c_{m}-c_{im}\right)-\left(\theta_{im}\mu_{l,im}+\left(1-f\right)\rho_{b}K_{d}\mu_{s,im}\right)c_{im}+\theta_{im}r_{l,im}\left(x\right)+\left(1-f\right)\rho_{b}r_{s,im}\left(x\right),$$

where the subscripts *m* and *im* refer to the mobile and immobile liquid regions, respectively,$\;J_{w}=v\times \theta =v_{m}\times \theta_{m}$ is the volumetric water flux density, *f* represents the fraction of adsorption sites that equilibrates with the mobile liquid phase, and a is the first-order mass transfer coefficient governing the rate of solute exchange between the mobile and immobile liquid regions. θ is equal to $\theta_{m}+\theta_{im}$. $\mu_{l,m}$ and $\mu_{l,im}$ are first-order decay coefficients for the mobile and immobile liquid phases, respectively; $\mu_{s,m}$ and $\mu_{s,im}$ are first-order decay coefficients for the mobile and immobile adsorbed phases, respectively; $r_{l,m}$ and $r_{l,im}\;$ are zero-order production for the mobile and immobile liquid phases, respectively; and $r_{s,m}$ and $r_{s,im}$ are zero-order production terms for the mobile and immobile adsorbed phases, respectively.

## 3. Experiments

### 3.1. Experimental Device

**Figure 1:**
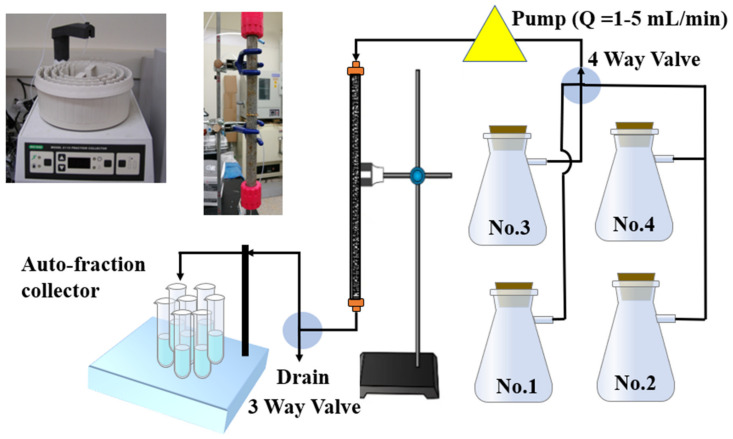
ADE experimental device in this work (schematic diagram).

Figure 1 shows the dynamic column experimental device used in this experiment. The experimental devices consisted of a peristaltic pump (MASTERFLEX L/S, Cole-Parmer Instrument Co., Barrington, IL, USA ), a high-performance silica glass cylinder (modified type, Shanghai SuKe Industrial Co., Ltd. Shanghai, China), an automatic liquid sample collector (MODEL 2110, Bio-Rad Laboratories, Inc., Hercules, CA, USA), and four reservoirs: namely, No. 1, synthetic groundwater (SGW); No. 2, radiotracer (HTO); No. 3, I^−^; and No. 4, IO_3_^−^ and glass microfiber filters (0.4 mm thickness) with a pore size of 0.7 um (GF/F, Associated Design & Manufacturing Co., Alexandria, VA, USA). Table 1 lists the parameters of the filled granite, the composition of the synthetic groundwater, and the size of the column. The inner diameter of the experimental column was 1.6 cm, and the length was 30 cm. A total of 85.40 ± 0.50 g crushed granite was filled, with a density of 1.45 ± 0.05 g/cm^3^ and a porosity of 0.47 ± 0.03. In order to provide a stable experimental flow rate, the device was also equipped with a pressure sensor and switch drain valves.

### 3.2. Rocks and Liquids

A discrete granitic island surrounding Fujian Province and local synthetic GW were sampled and investigated in previous studies [6,10]. All granite samples were crushed with a grinder to a particle size of 1 mm. Before filling the soil column to crush granite samples, all granite samples were washed three times with deionized water, dried at 100 ± 10 °C for 12–48 h, and stored in a vacuum dryer for future use.

### 3.3. Mineral Composition and Elemental Analysis

A mineral analysis of granite in rock was conducted using X-ray diffraction (XRD) as previously described. XRD spectra and micro-polar microscopy images showed that the main minerals included quartz, plagioclase, K-feldspars, amphibole, and biotite [31,32]. In addition, the crushed granite samples (~2 g) were analyzed under scanning electron microscopy coupled with energy dispersive spectrometry (SEM–EDS) with an accelerating voltage of 20 kV and a current of 10 μA. EDS was used to analyze the major elements of the granite samples.

### 3.4. Pre-Equilibrium: Water Saturation in Column

In order to simulate the migration behavior of nuclides under aquifer conditions, it was necessary to saturate the crushed granite column. The valve was opened to make the groundwater flow into the column slowly at a flow rate of 3 mL/min. During the water filling, 10 mL of effluent from each column was extracted every 30 min. The concentrations of K, Ca, Mg and Na in the effluent were measured by ICP-OES. When the concentration changed within 5% of the corresponding liquid phase concentration, it indicated that water saturation had been reached.

### 3.5. Nonreactive Tests of HTO

In order to characterize the internal structure of the crushed granite column, the dispersion and effective porosity were obtained by convection dispersion experiments with nonreactive tracer (HTO). By regularly collecting the effluent at different flow rates, the HTO content in the effluent was analyzed and measured, and the data were used to draw the penetration curve BTC. The water circulation was divided into two stages. The first stage was the manual operation stage, and its basic process was as follows. As shown in Figure 1, set water circulation under different flow rate conditions, 3 PV (about 2100 Bq) each time, including up/down flooding. The liquid sample was collected by an automatic sampler, and a 5 mL aliquot of the effluent was sampled to measure HTO activities with a liquid scintillation counter (Packard 3170 AB/TR, Shelton, CT, USA). During the first upflooding process, both control valves were open to reservoir No. 2, and the flow rates through the column were controlled at approximately 5.0 ± 0.1, 3.0 ± 0.1, and 1.0 ± 0.1 mL/min. After 3 PV of the liquid phase passed through the column in the upflooding process, the HTO was flushed out from the column in a downflooding process where switch valves were open to reservoir No. 1 (GW). The effluent was collected using an auto-fraction collector every 60, 100, and 300 s. Table 2 shows that this up/downflooding process was set up and repeated two more times. A 10 mL aliquot of the sample was mixed with 10 mL of scintillation cocktail in a 20 mL polyethylene counting vial. All chemicals used in the experiments were of analytical purity, and de-ionized water (DIW) was used throughout the experiments.

### 3.6. Reactive Tests of I^−^ and IO_3_^−^ (Anion Exclusion)

The transport model was calibrated/validated by designing ADE experiments at different flow rates [33]. I^−^ and IO_3_^−^, as well as disodium potassium iodide (KI) and potassium iodate (KIO_3_), were injected into the column as stable isotope tracers after the HTO in the column was completely washed out. The corresponding concentrations (C(t)) of I^−^ and IO_3_^−^ were determined via ion chromatography (IS-600, Thermo Fisher Scientific Inc., Waltham, MA, USA) for I speciation (I^−^ or IO_3_^−^), and the iodine concentrations in the solution were measured with an induced coupled plasma optic emission spectrometer (iCAP 7000, Thermo Fisher Scientific Inc., Waltham, MA, USA). Table 2 lists the results of a multi-stage ADE column test, which included a series of upflooding/downflooding processes, for I^−^ and IO_3_^−^. During the advection–dispersion experiments (ADEx) period for I^−^ and IO_3_^−^, the frequency of collection depended on the flow rate, that is, every 60 s for approximately 3 mL/min. The corresponding concentrations (C(t)) of I^−^ and IO_3_^−^ in the effluent were then determined, and the respective breakthroughs were plotted for analysis. To ensure that the ADEx was finished, the upflooding process was stopped when the relative concentration (C/C_0_) reached approximately 1.0, and the downflooding process was stopped when C/C_0_ decreased to 0.05–0.1.

### 3.7. Mathematical Model and Parameter Estimations

STANMOD is a Windows-based computer software package for evaluating solute transport in porous media using analytical solutions of the convection-dispersion solute transport equation. The software package includes a modified and updated version of the CXTFIT code for estimating solute transport parameters using a nonlinear least-squares parameter optimization method. Residuals are a measure of the degree to which experimental results (*Ce*) deviate from the predicted values (*Cp*), *N* is the number of experimental data, and the root mean square error (RMSE) is defined as

(11) $$RMSE=\sqrt{\frac{\sum_{i}^{N}{\left(C_{p}-C_{e}\right)}^{2}}{N}}.$$

The HYDRUS-1D program numerically solves the Richards equation for variably saturated water flow and advection-dispersion type equations for heat and solute transport. It uses a numerical solution for numerical fitting. The transport equations include provisions for nonlinear nonequilibrium reactions between the solid and liquid phases. In addition, physical nonequilibrium solute transport can be accounted for by assuming a two-region, dual-porosity type formulation that partitions the liquid phase into mobile and immobile regions. As part of the inverse solution, HYDRUS produces a correlation matrix that specifies the degree of correlation between the fitted coefficients. An important measure of the goodness of fit is the $\;r^{2}\;$ value for regression of the observed $\hat{y_{i}}$ versus fitted *y_i_* values:

(12) $$r^{2}=\frac{\left[\sum w_{i}\hat{y_{i\;}}y_{i}-\frac{\sum \hat{y_{i\;}}\sum y_{i}}{\sum w_{i}}\right]}{\left[\sum w_{i}{\hat{y_{i\;}}}^{2}-\frac{{\left(\sum \hat{y_{i\;}}\right)}^{2}}{\sum w_{i}}\right]\left[\sum y_{i}{}^{2}-\frac{\sum y_{i}{}^{2}}{\sum w_{i}}\right]}.\;$$

The $r^{2}$ value is a measure of the relative magnitude of the total sum of squares associated with the fitted equation; a value of 1 indicates a perfect correlation between the fitted and observed values. where $w_{i}$ is weighting factor for the two overlapping regions of the model,$\;\hat{y_{i\;}}$ is fitting value,$\;y_{i}$ is observed value.

## 4. Results and Discussion

### 4.1. Elemental Analysis by SEM–EDS

**Figure 2:**
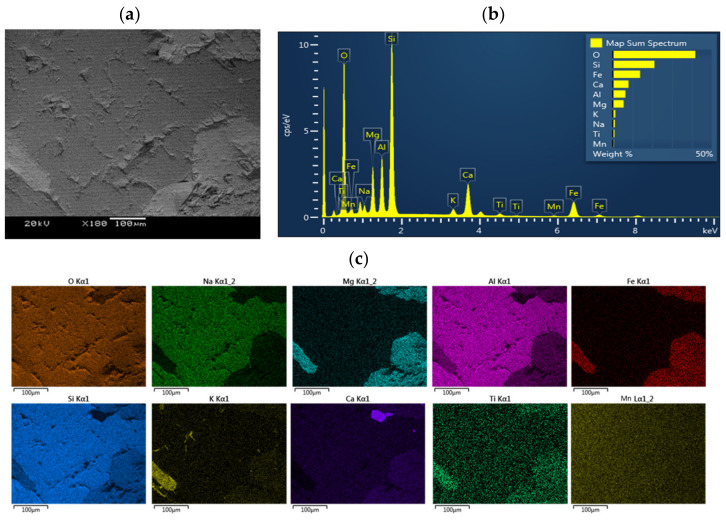
The SEM photos in granite and the EDS mapping of surface of granite obtained at 10 μm spatial resolution. (**a**) SEM (**b**) EDS spectrum (**c**) elemental mapping.

XRD, micro-X-ray computed tomography, polar microscopy, and analysis in previous works [31,32,34] showed that granite contains crystalline particles because it is igneous rock. In addition, we obtained and compared the corresponding images of SEM-EDS and elemental mapping analysis (Figure 2). Comparison of the mapping area and mineral components in granite showed that O, Si, Al, Mg Fe, K, Na, Ti, Mn, and Ca are the major mineral components, which agrees with previous studies that used different analysis methods, such as Rutherford backscattering spectrometry [34].

### 4.2. Experimental BTCs of HTO, I^−^, and IO_3_^−^

**Figure 3:**
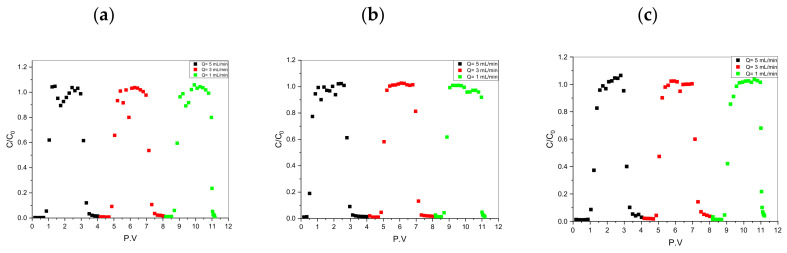
The ADE experimental breakthrough curves (BTCs). (**a**) HTO; (**b**) I^−^; (**c**) IO_3_^−^.

Before using I^−^ and IO_3_^−^, a nonreactive radiotracer (HTO) was applied to characterize the major physical transport processes in the proposed dynamic column system with a PV (1 PV = 28 mL). Figure 3 shows that the HTO, I^−^, and IO_3_^−^ BTCs at different flow rates reached 1 (*C*/*C_0_* = 1) from a series of sorption/desorption (up/downflooding) processes. Our experimental results indicated the absence of dead-end or dead pores to block the HTO, I^−^, and IO_3_^−^ pathways, and water was saturated in each pore space in the compacted granite powder.

It can be seen from the figure that since the injection method of solute is to continue to inject for a period of time and then stop the injection, all penetration curves are “s” type (divided into “s” type in the injection adsorption stage of the first half and “s” type in the desorption stage of the second half). Specifically, when the injection amount of solute reaches about one column pore volume, the ratio of solute activity concentration in the effluent quickly reaches the peak value, and then the concentration rapidly decreases to zero after continuous injection of clean water.

### 4.3. Fitting BTCs of Nonreactive Tracer (HTO)

**Figure 4:**
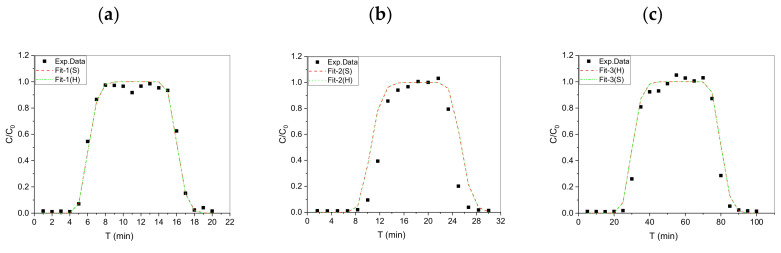
The HTO breakthrough curves (BTCs) at various flow rates. (**a**) 5.0 ± 0.1 mL/min; (**b**) 3.0 ± 0.1 mL/min; (**c**) 1.0 ± 0.1 mL/min.

The nonreactive tracer (HTO) was designed and applied in multi-stage ADEx to identify the reliability of our ADE device to build adequate reaction models through a calibration/validation process. Figure 4 (Fit-1 to -3) and Table 3 show that the equilibrium model in STANMOD- Fit (S) and HYDRUS-1D- Fit (H) were applied to fit the experimental data to obtain the parameters for HTO transport in the granite. The parameters (Fit-1 to -3) obtained by the two fitting methods showed similar results, indicating that the filling granite in the column showed good homogeneity. In addition, the highly reliable and accurate experimental data for HTO were identified according to the dispersivity (D_L_ = 0.270–0.289 cm), retardation factor R (=1), and distribution coefficient K_d_ (=0) in our ADE system by fitting with STANMOD and HYDRUS-1D.

### 4.4. Fitting BTCs of Anionic I^−^ and IO_3_^−^

**Figure 5:**
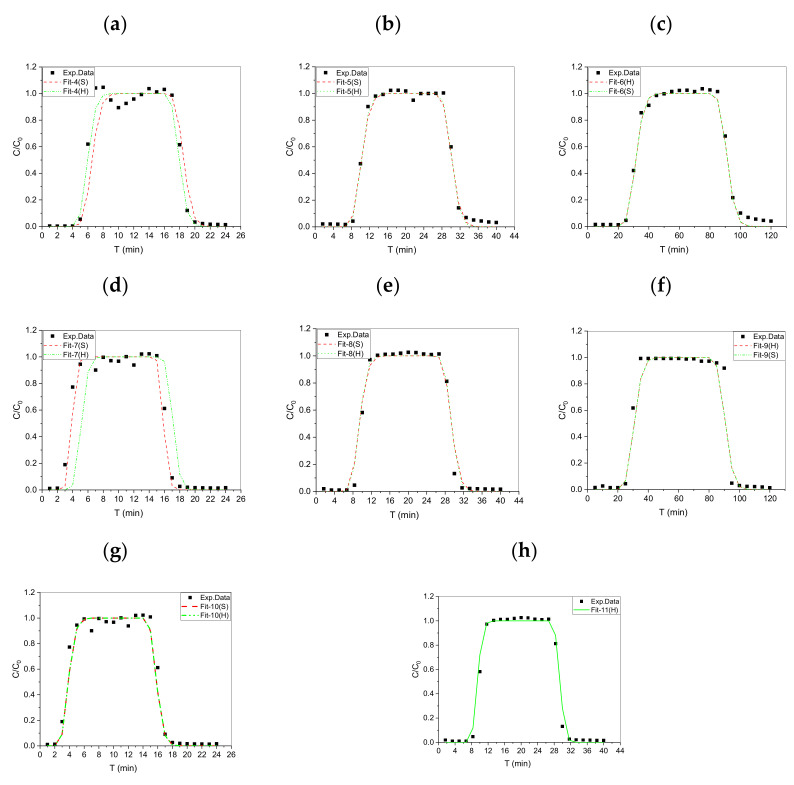
The experimental breakthroughs of I^−^ and IO_3_^−^ fitting curves. (**a**) IO_3_^−^: 5.0 ± 0.1 mL/min; (**b**) IO_3_^−^: 3.0 ± 0.1 mL/min; (**c**) IO_3_^−^:1.0 ± 0.1 mL/min; (**d**) I^−^: 5.0 ± 0.1 mL/min; (**e**) I^−^: 3.0 ± 0.1 mL/min; (**f**) I^−^: 1.0 ± 0.1 mL/min; (**g**) I^−^: 5.0 ± 0.1 mL/min; (**h**) I^−^: 3.0 ± 0.1 mL/min.

Figure 5 shows the BTCs of anionic I^−^ and IO_3_^−^ in the granite column at different flow rates, and only the BTCs of IO_3_^−^ (Fit-4 to -6) were similarly displayed and compared with the BTC of HTO. When the elution concentration ratio (C/C_0_) of IO_3_^−^ reached approximately 0.5 at 1 PV, R was almost equal to 1 and similar to HTO. Table 4 shows the fitting parameters of IO_3_^−^. The R (STANMOD) of IO_3_^−^ and distribution coefficient *K_d_* (HYDRUS-1D) were almost equal 0 (=0), showing the non-reactive or adsorptive behavior of IO_3_^−^ in granite.

As shown in Figure 5d and Table 5, the BTC C/C_0_ was less than 1 PV (Fit-7 and -8) of I^−^, and the R of I^−^ was lower than 1. An obvious anion repulsive effect occurred, which accelerated the I^−^ transport compared with HTO and IO_3_^−^ transport. In fact, for the BTC of I^−^, when the equilibrium model was used for fitting, when Q = 5.0 ± 0.1 mL/min, the analytical solution could be used for fitting to obtain a good effect. When the numerical solution was used for fitting, the fitting effect was poor. This phenomenon did not occur when fitting the penetration curve with Q = 1.0 ± 0.1 and 3.0 ± 0.1 mL/min.

In terms of I^−^ fitting parameters, the R of I^−^ obtained by fitting 5.0 ± 0.1 mL/min (Fit-7) and Q = 3.0 ± 0.1 (Fit-8) curves with analytical solution was less than 1, possibly indicating obvious anion exclusion. After fitting with the numerical solution in Table 5, the *K_d_* of the curve with Q = 5.0 ± 0.1 mL/min was greater than 1 by default (Fit-7). Thus, the fitting correlation was not good enough (*r^2^* = 0.77) compared with Fit-8 and -9 (*r^2^* > 0.95). The *K_d_* of I^−^ Q = 3.0 ± 0.1 mL/min was smaller than that of HTO, and I^−^ transport in granite could not be explained by fitting the equilibrium models (STANMOD and HYDRUS−1D).

Table 6, Fit-10 and 11 (*r^2^* > 0.95) show that the two-region nonequilibrium model was developed [33] and applied in this work to fit the BTC curves of Q = 5.0 ± 0.1 and 3.0 ± 0.1 mL/min (STANMOD) and Q = 5.0 ± 0.1 mL/min (HYDRUS-1D) and understand the I^−^ transport in granite. The fitting parameters showed that the water contents of immobile liquid phases ($\theta_{im}$) increased with increasing flow velocity (Fit-10), indicating that the anion exclusion effect was more obvious. In addition, the $\theta_{im}$ was larger than the analytical solution possibly because of the numerical algorithm.

**Figure 6:**
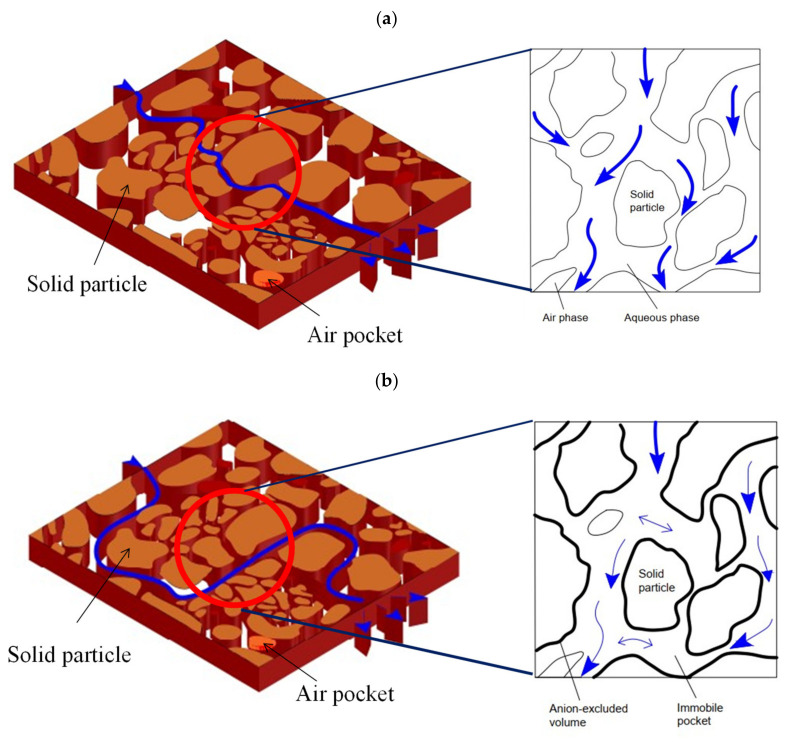
Schematic model of HTO and I^−^ transport. (**a**) HTO; (**b**) I^−^.

The migration behavior of HTO and I^−^ in the same medium was compared microscopically to explain the influence of anion exclusion of I^−^. Figure 6a describes the migration of HTO in the groundwater environment. As shown in the figure, HTO can pass through all water channels under the action of convection dispersion and finally fill the whole medium. Figure 6b shows the migration of I^−^ in the groundwater environment. According to the “electric DDL theory”, the anion concentration in a single pore increases exponentially with the distance from the pore wall. When I^−^ migrates in a wide water passage, it will converge to the center due to anion exclusion and will not pass through a narrow passage, forming “immobile regions”. Finally, I^−^ will select the channel with a wide water surface to migrate, resulting in acceleration.

**Figure 7:**
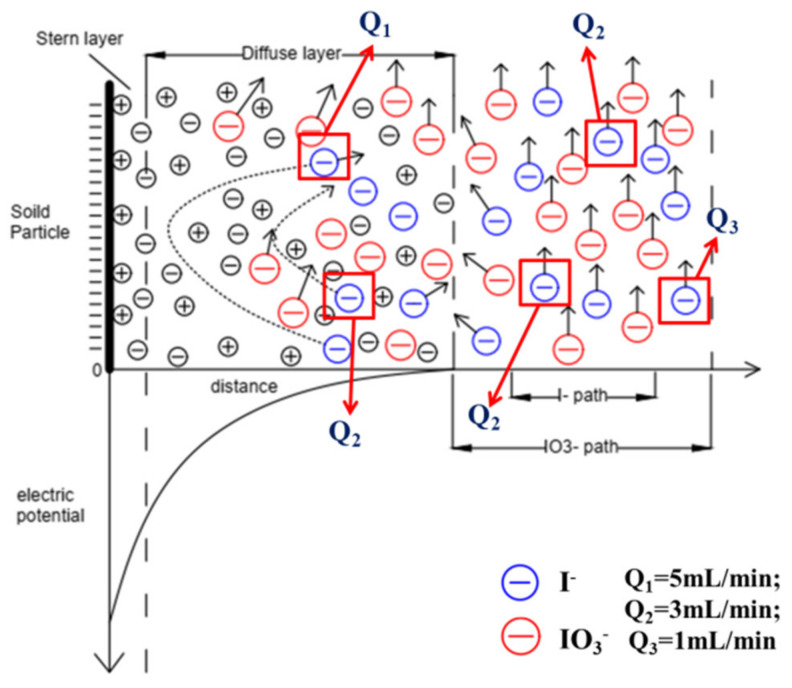
Schematic model of I^−^ and IO_3_^−^ transport at various flow velocities.

Figure 7 shows a comparison of the migration behaviors of I^−^ and IO_3_^−^ under different flow rates. According to the theory of the “electric double layer model”, a negative ion layer forms when the dielectric surface is negatively charged, and a positive ion layer forms outside due to electrical attraction. The closer it is to the dielectric surface, the more negative its potential becomes (the more obvious the repulsion). Comparison of the ion radii of I^−^ and IO_3_^−^ showed that the ion radius of I^−^ (3.31 Å) was smaller than that of IO_3_^−^ (3.74 Å) [35]. Therefore, I^−^ can pass through the diffusion layer and become closer to the medium surface during migration. At the same time, the greater the migration speed of ions, the greater the kinetic energy generated, the closer the energy to the medium surface, the stronger the anion exclusion effect, and the more obvious the acceleration during migration. However, it is difficult for IO_3_^−^ to become close to the medium surface because of its large ion radius and weak anion exclusion. Thus, the acceleration during migration was not obvious.

## 5. Conclusions

The transport behavior of HTO, I^−^, and IO_3_^−^ in granite was studied by ADE column experiments. Comparison of the BTCs of HTO, I^−^, and IO_3_^−^ under different flow rates showed that the anion exclusion effect occurred in I^−^at Q = 5.0 ± 0.1 mL/min during fitting STANMOD and HYDRUS-1D when R < 1. The key parameters of HTO, I^−^, and IO_3_^−^ were obtained by fitting the equilibrium and nonequilibrium models of the analytical method (STANMOD) and numerical inversion (HYDRUS-1D). The conclusions are as follows:

1. The BTCs for HTO were symmetrical at various flow rates (1.0 ± 0.1–5.0 ± 0.1 mL/min), and no significant differences were found in the accessible porosity and dispersivity values of HTO. Therefore, the experimental apparatus was highly reliable, and the granite samples filled and compacted in the column were almost homogeneous.
2. Comparison with the BTCs of HTO, I^−^, and IO_3_^−^ at different flow rates showed that obvious anion exclusion only occurred in I^−^ transport by increasing the flow rate from 1 to 5. In fact, the anion exclusion (R < 1) only occurred for I^−^ at a flow rate of 5 mL/min, and a relative Coulomb’s repulsive force may be caused by the smaller hydration radius of I^−^(3.31 Å) than that of IO_3_^−^(3.74 Å) according to electric DDL theory.
3. The equilibrium and nonequilibrium transport models were used and compared to identify the mobile/immobile zones in the compacted granite column. The anion exclusion effect depended on the immobile zones in the column. In sum, the nonequilibrium model can well characterize the immobile regions of anion exclusion, which is obviously proportional to the value of immobile regions.
4. Since the experiment was conducted in laboratories, the environmental conditions were quite different from the actual environment. Whether the research results can be applied to the actual plant site needs to be demonstrated. It is recommended to carry out field experiments as soon as possible.

## Figures and Tables

**Table 1:** The experimental crushed granite and synthetic groundwater (SGW) in this study.

Item	Granite	SGW (M)			
Location	Fujian Province, China	Ca^2+^	3.76 × 10^−4^	F^−^	1.85 × 10^−4^
Particle Size (mm)	<1	Mg^2+^	6.38 × 10^−5^	HCO_3_^−^	1.21 × 10^−3^
Length (cm)	30.0	Na^+^	1.57 × 10^−3^	pH	8.0 ± 0.2
Diameter (cm)	1.6	K^+^	9.22 × 10^−5^	Eh	220 ± 20
Weight (g)	85.40 ± 0.50	Cl^−^	9.77 × 10^−4^	T(°C)	20 ± 2
bulk density (g/cm^3^)	1.45 ± 0.05	SO_4_^2^^−^	1.05 × 10^−4^	I (M)	3.11 × 10^−3^
Porosity	0.47 ± 0.03	I: Ionic Strength			

**Table 2:** ADE experimental process under different flow rates.

Batch		No. 1	No. 2	No. 3	Remark
	RN	HTO: 50 Bq/mL (A_0_)	I^−^ 7.78 (C_0_: ppm)	IO_3_^−^ 5.41 (C_0_: ppm)	
Flow Rate (mL/min)					
5.0 ± 0.1		U	U	U	U: up-flooding D: down-flooding V_0_ = 2000 mL
		D	D	D	
3.0 ± 0.1		U	U	U	
		D	D	D	
1.0 ± 0.1		U	U	U	
		D	D	D	

**Table 3:** The parameters for fitting HTO BTCs.

	STANMOD (S)			HYDRUS-1D (H)		
HTO	Fit 1	Fit 2	Fit 3	Fit 1	Fit 2	Fit 3
Q (mL/min)	5.0 ± 0.1	3.0 ± 0.1	1.0 ± 0.1	5.0 ± 0.1	3.0 ± 0.1	1.0 ± 0.1
*D_L_* (cm)	0.268	0.331	0.268	0.270	0.270	0.269
*R*	1.11	1.07	1.29	1.17	1.21	1.16
*K_d_* (mL^3^/g)	=0	=0	=0	=0	=0	=0
RMSE/*r^2^*	4.05 × 10^−2^	1.62 × 10^−1^	8.58 × 10^−2^	0.99	0.90	0.97
$\;\;\;\;\;\;\;\;\;\;\;\;\;\;\;\;\;\;\;\;\;\;R=1+\frac{\rho_{b}K_{d}}{\theta }$; Bulk Density = 1.45 ± 0.05 g/cm^3^; porosity = 0.47 ± 0.03						

**Table 4:** The parameters for fitting IO_3_^−^ BTCs.

**STANMOD (S)**	**No**	**Flow Rate (mL/min)**	***v* (cm/min)**	***D* (cm^2^/min)**	***R***	***D_L_* (cm)**	**RMSE**
	**Fit 4**	5.0 ± 0.1	5.41	1.45	1.19	0.268	0.14 × 10^−1^
	**Fit 5**	3.0 ± 0.1	3.02	1.00	1.04	0.331	0.15 × 10^−2^
	**Fit 6**	1.0 ± 0.1	1.27	0.34	1.34	0.268	0.15 × 10^−2^
**HYDRUS-1D (H)**	**No**	**Flow Rate (mL/min)**	***Ks* (cm/min)**	***K_d_* (mL^3^/g)**	***D_L_* (cm)**	***r^2^***	
	**Fit 4**	5.0 ± 0.1	2.488	0.050	0.270	0.98	
	**Fit 5**	3.0 ± 0.1	1.492	0.044	0.270	0.99	
	**Fit 6**	1.0 ± 0.1	0.498	0.069	0.269	0.99	

**Table 5:** The parameters for fitting I^−^ BTCs (equilibrium model).

**STANMOD** **(S)**	**No**	**Flow Rate (mL/min)**	***v* (cm/min)**	***D* (cm^2^/min)**	***R***	***D_L_* (cm)**	**RMSE**
	**Fit 7**	5.0 ± 0.1	5.41	1.45	0.71	0.268	0.55 × 10^−2^
	**Fit 8**	3.0 ± 0.1	3.02	1.00	0.96	0.331	0.37 × 10^−2^
	**Fit 9**	1.0 ± 0.1	1.27	0.34	1.31	0.268	0.87 × 10^−2^
**HYDRUS-1D (H)**	**No**	**Flow Rate (mL/min)**	***Ks* (cm/min)**	***K_d_* (mL^3^/g)**	***D_L_* (cm)**	***r^2^***	
	**Fit 7**	5.0 ± 0.1	2.488	=0	0.270	0.77	
	**Fit 8**	3.0 ± 0.1	1.492	=0	0.270	0.99	
	**Fit 9**	1.0 ± 0.1	0.498	=0	0.269	0.96	

**Table 6:** The parameters for fitting I^−^ BTCs (non-equilibrium model).

**STANMOD** **(S)**	**No**	**Flow Rate (mL/min)**	***v*** **(cm/min)**	***D*** **(cm^2^/min)**	$\theta_{m}$	$\theta_{im}\;$	***D_L_*** **(cm)**	***α* (min^−1^)**	**RMSE**
	**Fit 10**	5.0 ± 0.1	7.66	3.95	0.368	0.067	0.516	2.70	0.54 × 10^−2^
	**Fit 11**	3.0 ± 0.1	3.17	0.52	0.471	0.019	0.164	3.85	0.27 × 10^−2^
**HYDRUS-1D (H)**	**No**	**Flow rate (mL/min)**	***K_s_*** **(cm/min)**		$\theta_{m}$	$\theta_{im}$	***D_L_*** **(cm)**	***A* (min^−1^)**	***r^2^***
	**Fit 10**	5.0 ± 0.1	3.279		0.358	0.102	0.530	3.04	0.98

## Data Availability

Not applicable.
